# Supplementary material for: A spatial map of hepatic mitochondria uncovers functional heterogeneity shaped by nutrient-sensing signaling
Source: Nat Commun. 2024 Feb 28;15:1799. doi: 10.1038/s41467-024-45751-9 (PMC10902380; doi:10.1038/s41467-024-45751-9)
Supplement: Supplementary file 1 — Supplementary Information [file 41467_2024_45751_MOESM1_ESM.pdf]

A spatial map of hepatic mitochondria *Kang et al.*

Supplementary Figures

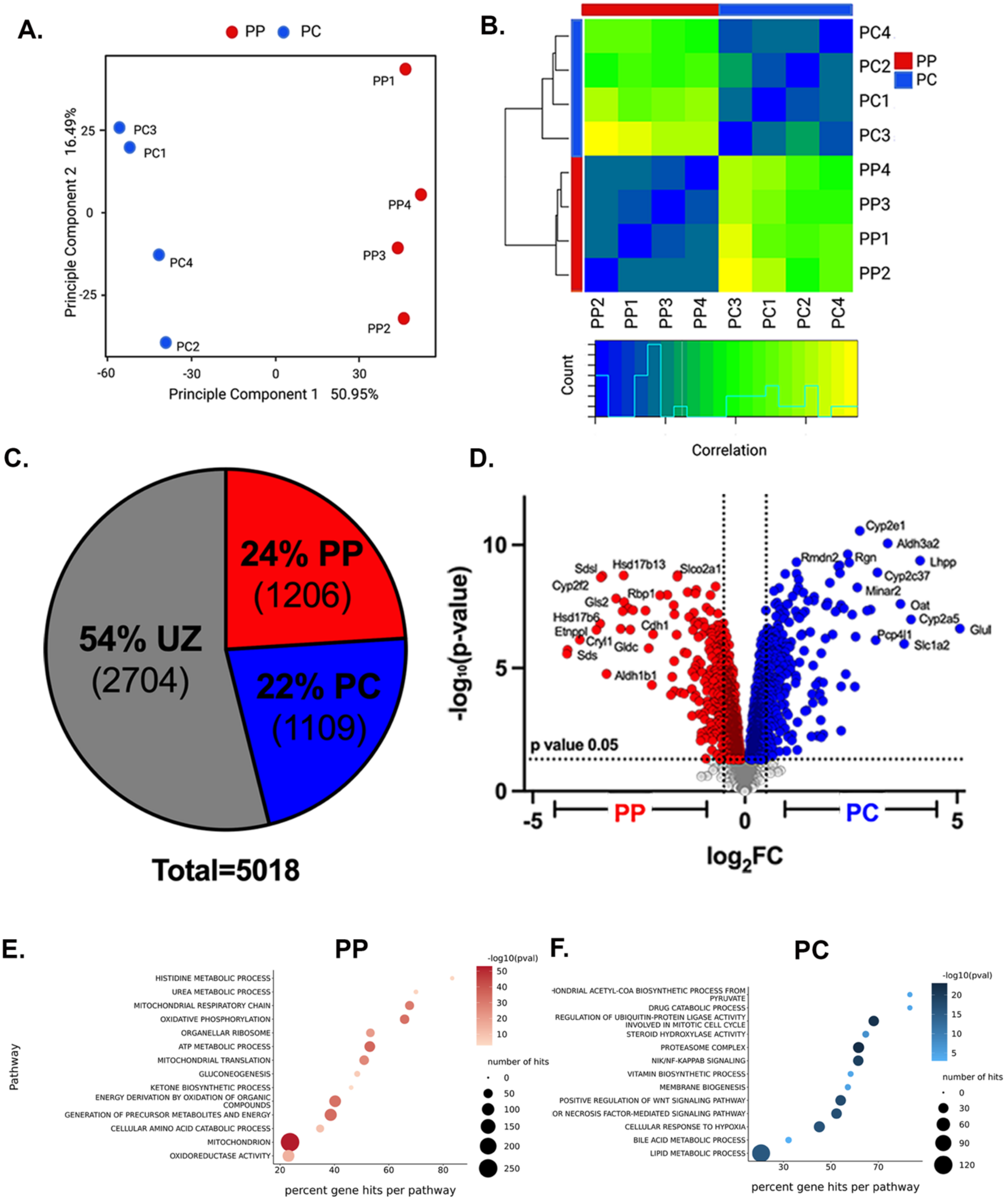

Supplementary Figure 1. Comparative proteomics of spatially sorted hepatocytes. (A) Principal component analysis (PCA) and frequency histogram of spatially sorted hepatocytes analyzed with mass spectrometry. (B) Correlation Matrix Heatmap of proteomics data. (C) Pie chart depicting the percentage of PP, PC, and UZ proteins based on p-value (0.05). (D) Volcano plot showing the *PC* to PP log_2 fold-change (x-axis) and the $-log_{10}$ p-value (y-axis) for identified proteins. (E and F) GO enrichment analysis of the proteomics data in the spatially sorted cell.

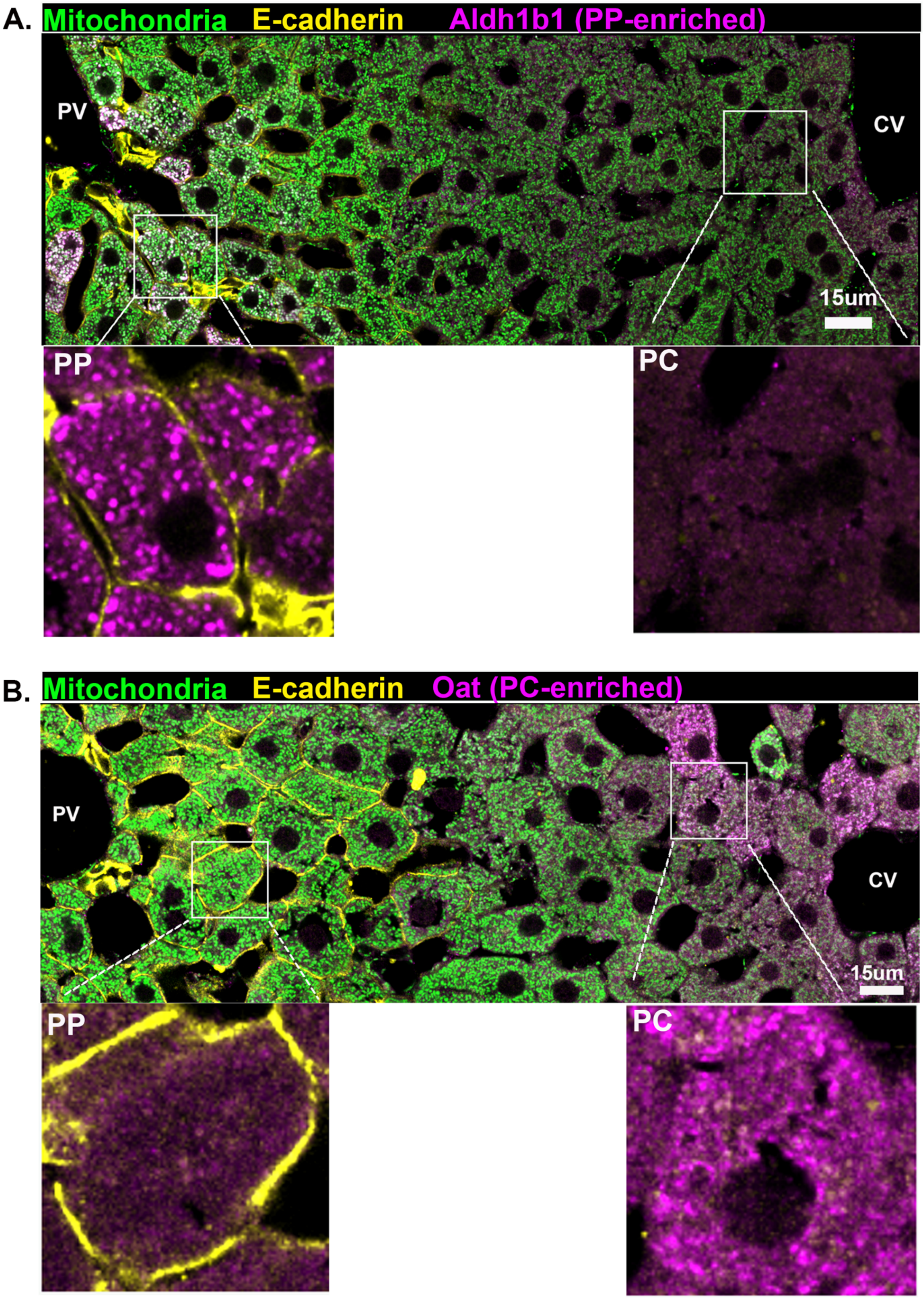

Mitochondria zonation. Kang *et al.*

Supplementary Figure 2. Immunofluorescence of representative PP and PC mitochondrial proteins. (A) Confocal image of the liver lobule from Mito-Dendra2 mice (green) stained with E cadherin to label PP regions (yellow) and Aldh1b1, a PP mitochondrial protein (magenta). Magnified insets show the overlay of E-cadherin and Aldh1b1 only. Scale bar: 15 μm. (B) Confocal image of the liver lobule from Mito-Dendra2 mice (green) stained with E-cadherin to label PP regions (yellow) and Oat, a PC mitochondrial protein (magenta). Magnified insets show the overlay of E-cadherin and Oat only. Scale bar: 15 μm.

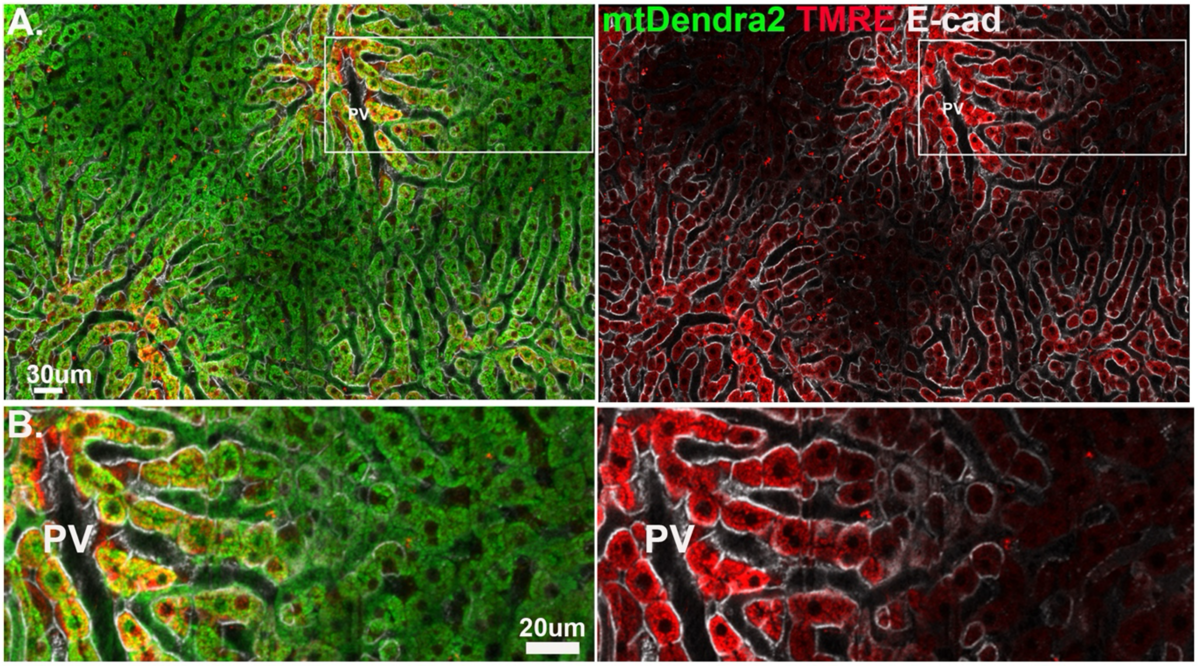

Supplementary Figure 3. E-cadherin-positive hepatocytes display higher membrane potential. (A-B) Intravital microscopy of the hepatic lobule in Mito-Dendra2 mouse labeled with TMRE (red), and E-cadherin (white). (A) Low magnification of the hepatic lobule. Scale bar: 30 μm. (B) Close up on the PP-PC axis. Scale bar: 20 μm.

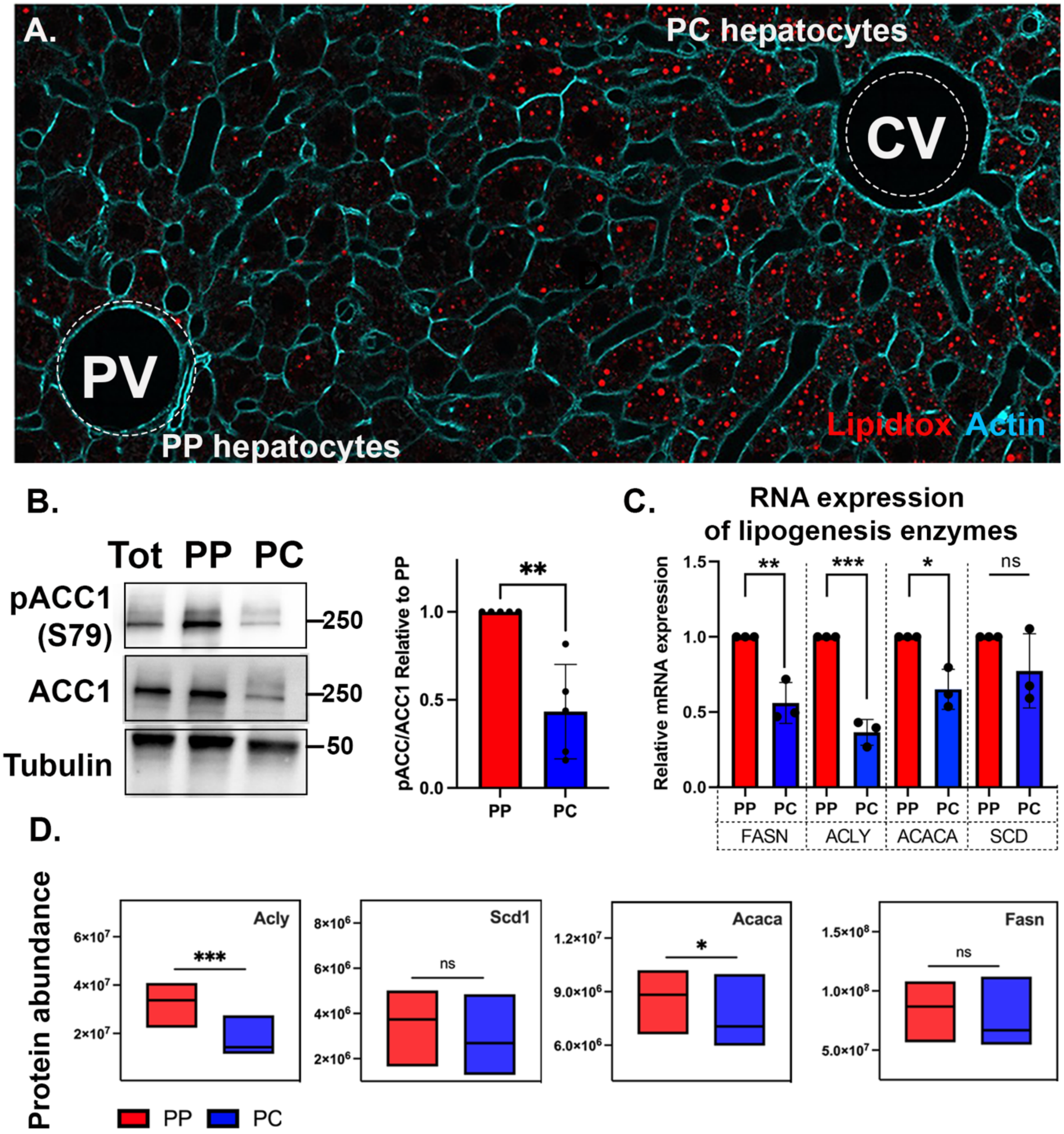

Supplementary Figure 4. Lipid synthesis is pericentral in the murine liver. (A) Confocal image of a liver section labeled with Lipidtox (lipid droplets; red) and phalloidin (actin; cyan), showing the non-uniform distribution of lipid droplets across the PP-PC axis. (B) Representative immunoblot and quantification of acetyl-CoA carboxylase 1 (ACC1, Acaca) relative phosphorylation (S79) in spatially sorted hepatocytes. Data presented as mean ± SD from n=5 independent experiments.

Statistical significance was calculated using two-tailed unpaired Student's t-test. Statistical significance is denoted as **p=0.002. (C) RNA levels of key lipogenesis enzymes in spatially sorted hepatocytes. Data presented as mean ± SD from n=3. Statistical significance was calculated using two-tailed unpaired Student's t-test; ns not significant (Scd1); *p=0.0105 (Acaca); **p=0.006 (Fasn); ***p=0.0002 (Acly). (D) Protein abundance of key lipogenesis enzymes identified by proteomics shown with floating bar graphs. The center line represents the mean value. Top and bottom of the box are extended to the maxima and minima, data from n=4 independent experiments. The proteomics data p values were calculated with Limma R package (v3.40.6); ns not significant (Scd1 and Fasn); *p=0.0102 (Acaca); ***p=0.0005 (Acly).

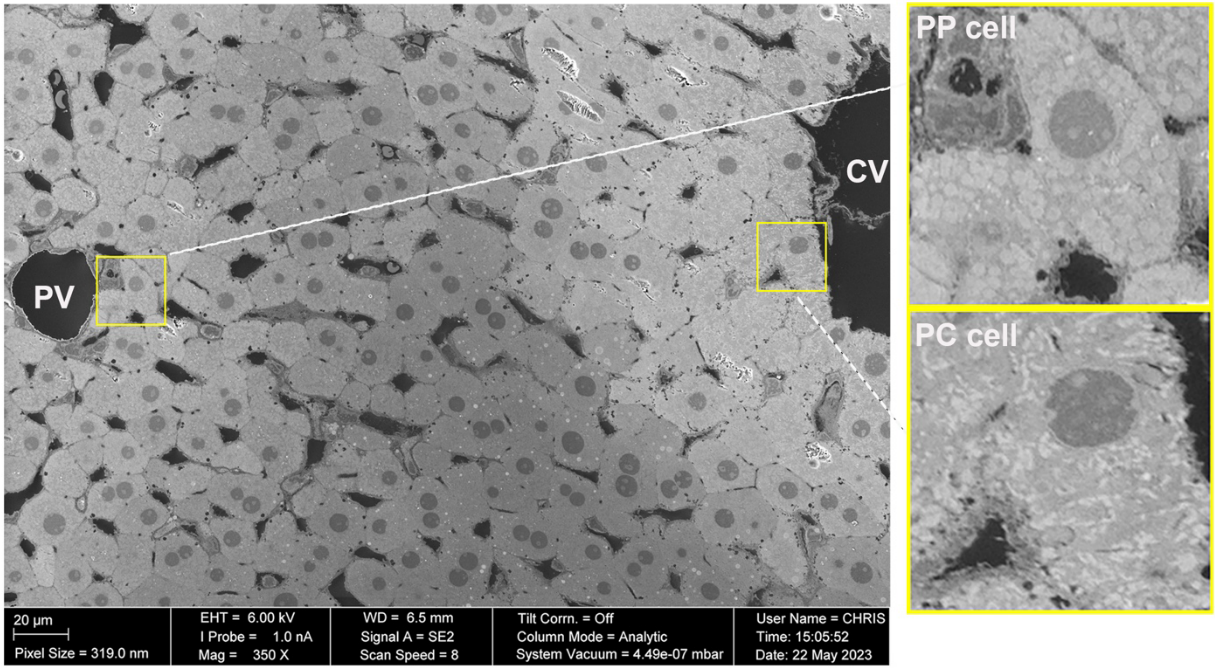

Supplementary Figure 5. Scanning Electron Microscopy (SEM) of the lobule highlighting regions of interest selected for FIB-SEM. SEM of the PP-PC axis in a liver section was performed to mark PP and PC regions for FIB-SEM. Insets show PP and PC hepatocytes where striking variations in mitochondrial morphology are observed.

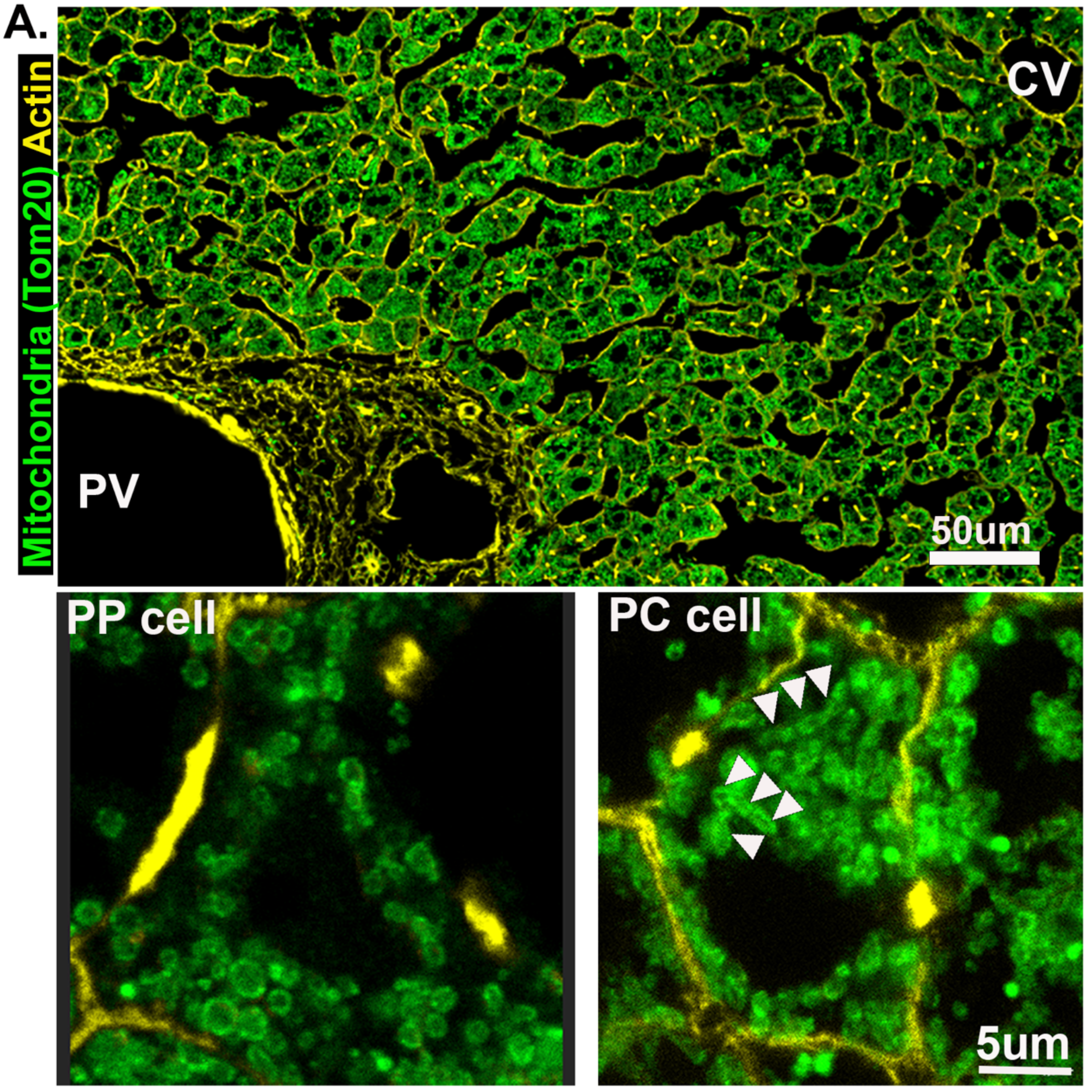

Supplementary Figure 6. Mitochondrial morphologies are conserved in the human liver. Confocal image of fixed human liver sections labeled with phalloidin (actin; yellow) and TOM20 (outer mitochondrial membrane; green). Scale bar: 50μm. Insets of representative PP and PC hepatocytes show distinct morphological features (arrows). Scale bar: 5μm.

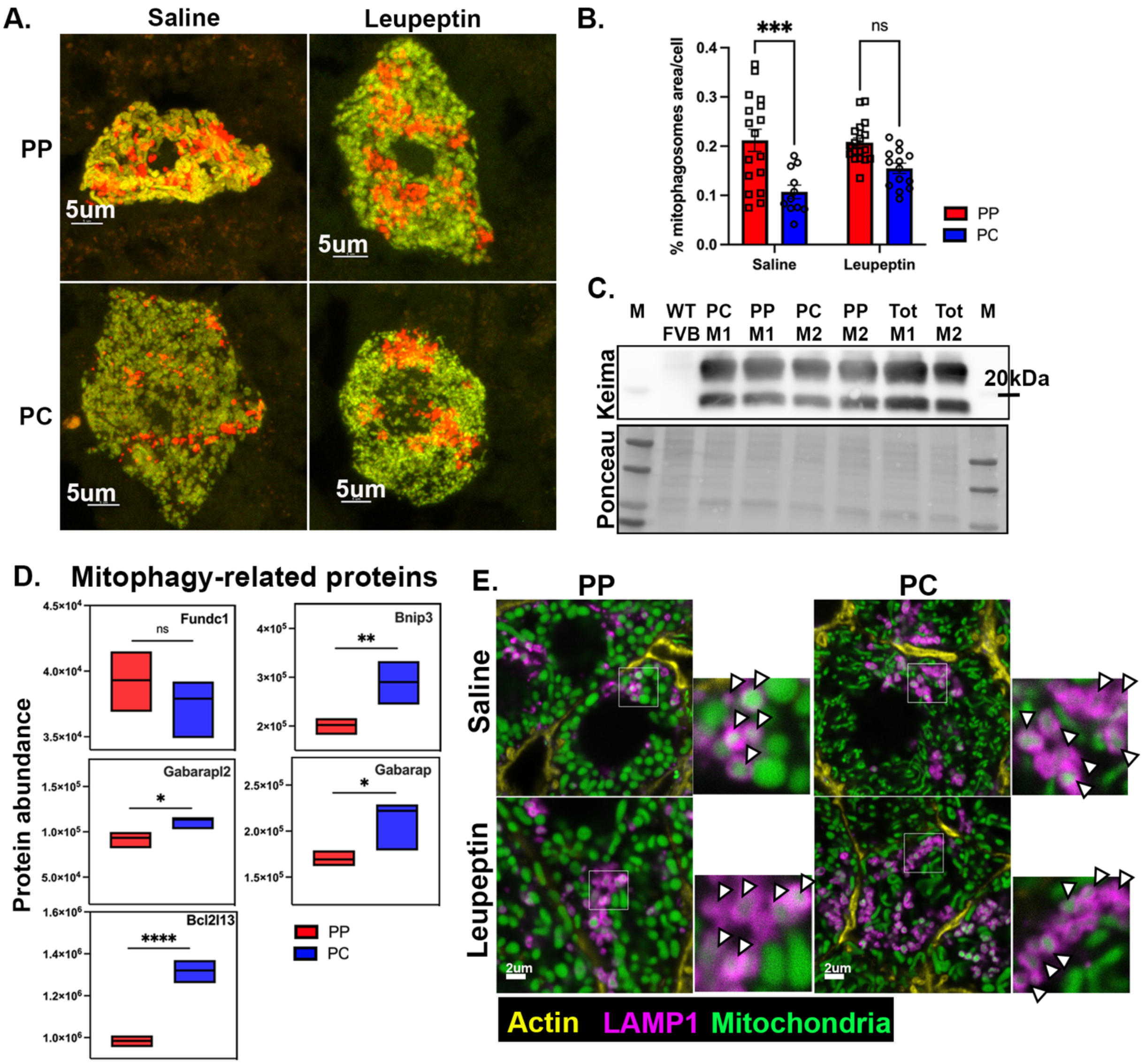

Supplementary Figure 7. PC mitochondria display higher mitophagy flux. (A) Confocal microscopy of liver sections from mice transduced with Cox8-EGFP-mCherry adenovirus. Representative PP and PC cells are shown from saline or leupeptin-treated mice. Scale bar: 5µm. (B) The Cox8-EGFP-mCherry reporter was used to measure mitophagy by calculating the percent of the mitophagosome area normalized to the whole cell area in saline or leupeptin-treated mice. Bar graph shows 15-20 cells quantified from 3 mice. (C) Keima protein expression in hepatocytes isolated from one wild-type mouse and two mtKeima mice (M1 and M2). Sorted (PP and PC) and total are shown for mtKeima only. (D) The abundance of mitophagy-related proteins identified by proteomics is shown with floating bar graphs. The center line represents the mean value. The

top and bottom of the box are extended to the maxima and minima, n=4 independent experiments. Statistical significance of proteomics data was calculated with Limma R package (v3.40.6). FUN14 domain-containing 1 (Fundc1; ns, not significant); Bcl2 interacting protein 3 (Bnip3; **p=0.002); GABA type A receptor-associated protein-like 2 (Gabarapl2; *p=0.0105); GABA type A receptor-associated protein like (Gabarapl; *p=0.014); BCL2-like 13 (Bcl2l13; ****p<0.0001). (E) Confocal images of PP and PC hepatocytes from Mito-Dendra2 mice (green), treated with saline or leupeptin and stained with the lysosomal marker LAMP1 (magenta) and actin (yellow). Arrowheads point to mitochondria (green) inside lysosomes (magenta).

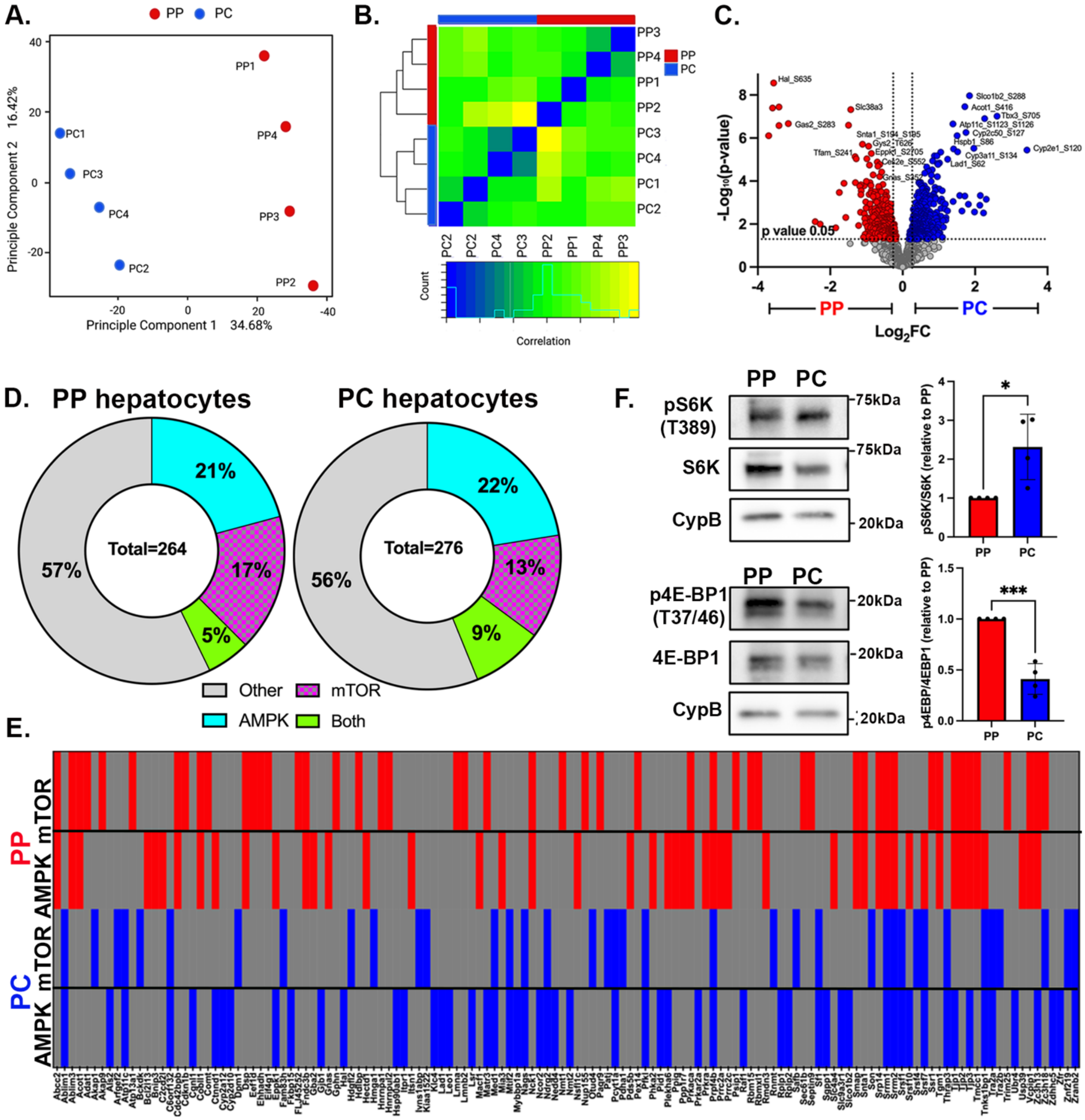

Supplementary Figure 8. Comparative phosphoproteome in spatially sorted hepatocytes. (A) Principal component analysis (PCA) and (B) Correlation Matrix Heatmap of the phosphoproteome data set. (C) Volcano plot showing the $\log_2$ *PC/*PP fold-change (x-axis) and the $-\log_{10}$ p-value (y-axis). (D) Group-based Prediction System (GPS 5.0) was used to predict mTOR and AMPK phosphorylation sequences. (E) Complete list of mTOR and AMPK substrates identified in PP and

PC. (F) Representative immunoblot and quantification of mTOR substrates pS6K (T389) and p4E-BP (T37/46) in spatially sorted hepatocytes. Data presented as mean ± SD from n=4 independent experiments. Statistical significance was calculated using two-tailed unpaired Student's t-test; *p=0.02 (S6K); ***p=0.0002 (4E-BP1).

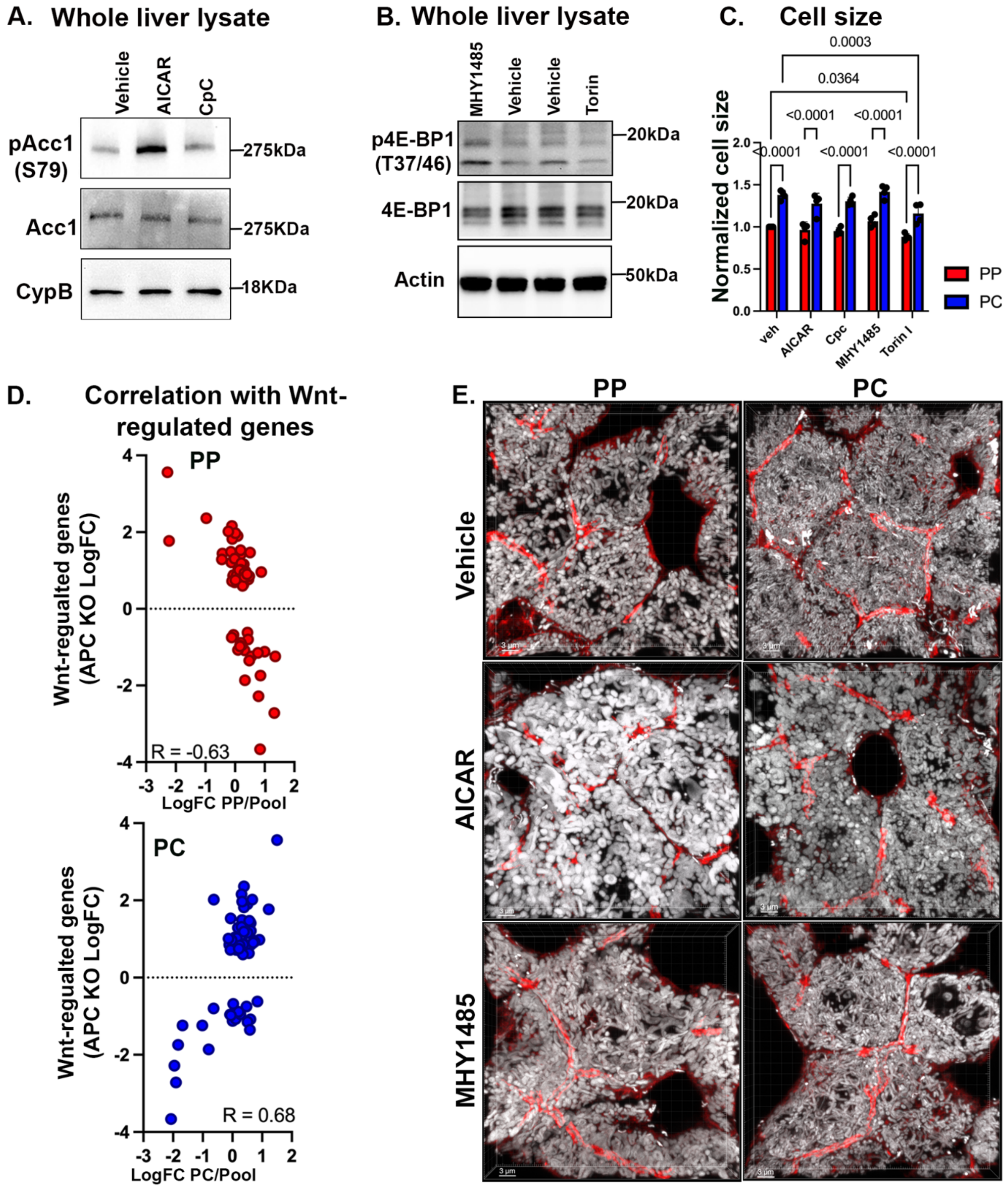

Supplementary Figure 9. Nutrient sensing signaling regulates mitochondrial heterogeneity. (A) Determination of AICAR and Compound C (CpC) concentrations for in vivo modulation of AMPK signaling. Drug efficacy was evaluated by Western blotting for pACC1 (S79) levels. (B) Determination of MHY1485 and Torin concentrations for in vivo modulation of mTOR signaling. Drug efficacy was evaluated by Western blotting for p4EBP1(Thr37/Thr46) levels. (C) Mice were treated with two doses of vehicle, AMPK inhibitor (Compound C) or activator (AICAR), or mTOR inhibitor (Torin I) or activator (MHY1485). Graphs show the evaluation of the drug effect on cell size measured by flow cytometry from n=4 independent experiments. Statistical significance was calculated using two-way ANOVA, and Fishers Least Significant Difference (LSD) test. (D) Correlation plot of Wnt-activated genes and PP or PC mitochondrial proteome. (E) Confocal z-stacks of hepatocytes from Mito-Dendra2 mice treated with vehicle, AICAR, or MHY1485. Mitochondria are shown in white, and phalloidin outlines hepatocytes in red. Scale bar= 3um.

Supplementary Tables:

PP

mod	motif	regexp	score	fg_match	fg_size	bg_match	bg_size	fg/bg	unadjusted_p-value	tests	adjusted_p-value
S	xxxxxxx_S_PxPxxxx	.......SP.P....	45.6	23	220	1	220	23	8.50E-07	301	2.60E-04
S	xxxxxxx_S_Pxxxxxx	.......SP......	22.33	88	220	19	220	4.6	3.40E-15	257	8.80E-13
S	xxxxxxx_S_DxExxxx	.......SD.E....	14.39	12	220	1	220	12	1.50E-03	178	2.30E-01
S	xxxxRxx_S_xxxxxxx	....R..S.......	6.62	61	220	34	220	1.8	1.20E-03	180	2.00E-01
T	xxxxxxx_T_Pxxxxxx	.......TP......	9.12	20	28	5	28	4	5.90E-05	23	1.40E-03

PC

mod	motif	regexp	score	fg_match	fg_size	bg_match	bg_size	fg/bg	unadjusted_p-value	tests	adjusted_p-value
S	xxxxxSx_S_Pxxxxxx	.....S.SP......	33.34	22	238	1	238	22	1.80E-06	265	4.80E-04
S	xxxxxxx_S_Pxxxxxx	.......SP......	11.48	68	238	17	238	4	4.00E-10	222	8.90E-08
S	Kxxxxxx_S_xxxxxxx	K......S.......	8.01	25	238	7	238	3.6	7.40E-04	172	1.20E-01
S	xxxxRxx_S_xxxxxxx	....R..S.......	7.44	69	238	35	238	2	1.10E-04	191	2.20E-02
S	xxxxxxx_S_Dxxxxxx	.......SD......	8.29	37	238	19	238	1.9	7.50E-03	135	6.40E-01
T	xxxxxxx_T_Pxxxxxx	.......TP......	7.82	17	27	4	27	4.2	3.10E-04	21	6.50E-03

Supplementary Table 1: Modification Motifs (MoMo) analysis

Protein	P-Zonation	P-site	Upstream regulator/kinase	Function
Acaca	PP	S29	Leptin, Raptor, BMP2, AMPK [1]	Lipogenesis
Akap1	PP	S101/S103/S104/S109	ADRB1, RIPK3<br>Insulin, LPA, LY294002, AMPK [2]	cAMP-PKA signaling
Apex1	PP	S18		mtDNA maintenance
Bcl2l13	PP	S387	Leptin	Mitophagy
Bnip3	PP	S79<br>S85<br>S88<br>T66	TSC2<br>ADRB1, TSC2<br>ADRB1, Rictor, STK3/4 [3]<br>JNK1/2 [4]	Mitophagy
Comt	PP	S261	Refeeding	Catechol metabolism
Ehhadh	PP	T543	Leptin	Fatty acid oxidation
Gpam	PP	S687<br>S694	Refeeding<br>Leptin, Refeeding	Phospholipid metabolism
Mtfr1	PP	T118		Fission
Mtfr1l	PP	S234/S235	Leptin, AMPK [5]	Fission
Nadk2	PP	S373		NAD biosynthesis and metabolism
Prkaca	PP	T198	PDK1 [6], PKACA, ADRB1, GPR107, Fasting	cAMP-PKA signaling
Rmdn3	PP	S46<br>S50	Leptin, PTH(1-34), PKA [7]<br>Insulin	Mitochondrial dynamics/contact sites
Slirp	PP	S105	Leptin	mtRNA stability
Tfam	PP	S241		mtDNA maintenance
Tomm70	PP	S94	ADRB1, Leptin, CK2α [8]	Protein import and sorting
Akap1	PC	T487		cAMP-PKA signaling
Bckdk	PC	S31	APN [9]	Amino acid metabolism
Bnip3	PC	S60	JNK1/2 [4]	Mitophagy
Coq9	PC	S81	Leptin, BMP2, SB202190	Coenzyme Q metabolism
Gpam	PC	S694	Leptin, Refeeding	Phospholipid metabolism
Hsd17b8	PC	S58		Lipid biosynthesis
Kars1	PC	S594		Protein translation

Mitochondria zonation. Kang *et al.*

Miga2	PC	S276		Fusion/contact sites
Mrps36	PC	S55/T59/S60		Mitochondrial translation
Mtif2	PC	S180		Protein translation
Nags	PC	S44		Amino acid metabolism
Nsun2	PC	S23	Rictor	RNA modification
Pdha1	PC	S293, S300	PDK1/3/4 [10], AMPKA2, AS184285, BCKDH E1α, DCA, Fasting, Hypoxia	Pyruvate metabolism
Tomm22	PC	S45	Insulin	Protein import and sorting

Supplementary Table 2: Extended table of mitochondrial phosphoproteins using PhosphoSitePlus [11].

ADRB1	Adrenoceptor Beta 1
AMPK	AMP-activated protein kinase
AMPKA2	Protein Kinase AMP-Activated Catalytic Subunit Alpha 2
APN	Aminopeptidase N
AS184285	Foxo1 (Forkhead Box O1) Inhibitor
BCKDH E1a	Branched Chain Keto Acid Dehydrogenase E1 Subunit Alpha
BMP2	Bone Morphogenetic Protein 2
CK2a	Casein Kinase 2 Alpha 2
GPR107	G Protein-Coupled Receptor 107
JNK1/2	c-Jun N-terminal kinase 1/2
LPA	Lipoprotein(A)
LY294002	Phosphoinositide 3-kinase inhibitor
PDK1-4	Pyruvate Dehydrogenase Kinase 1-4
PKA	Protein kinase A
PKACA	Protein Kinase CAMP-Activated Catalytic Subunit Alpha
PTH(1-34)	Selective activator of the parathyroid hormone receptor (PTH1R) signaling pathway
Raptor	Regulatory-associated protein of mTOR
Rictor	Rapamycin-insensitive companion of mammalian target of rapamycin
RIPK3	Receptor Interacting Serine/Threonine Kinase 3
SB202190	MAPK inhibitor

STK3/4	Serine/Threonine Kinase 3/4
TSC2	Tuberin

Supplementary Table 3:

**Antibodies**	**Source**	**Identifier**	**Dilution**
Recombinant Anti-Glutamine Synthetase antibody	AbCam	ab176562	1:1000
Recombinant Alexa Fluor® 647 Anti-TOMM20 antibody [EPR15581-54] - Mitochondrial Marker	AbCam	ab209606	1:100
Anti-LAMP1 antibody [EPR21026] (ab208943)	AbCam	ab208943	1:200
BD OptiBuild™ BUV395 Rat Anti-Mouse CD324/E-Cadherin	BD Biosciences	752475	1:150
PE/Cyanine7 anti-mouse/human CD324 (E-Cadherin) sntibody	Biolegend	147310	1:150
PE anti-mouse/human CD324 (E-Cadherin) antibody	Biolegend	147304	1:150
APC anti-mouse CD73 antibody	Biolegend	127210	1:150
Alexa Fluor® 647 anti-mouse/human CD324 (E-Cadherin) Antibody	Biolegend	147308	0.5 ug/g intravital; 1:100 IF
Alexa Fluor® 594 anti-mouse/human CD324 (E-Cadherin) Antibody	Biolegend	147306	1:100
Phospho-Acetyl-CoA Carboxylase (Ser79) antibody	Cell signaling	3661S	1:750
4EBP1 (Thr37/46) rabbit monoclonal antibody	Cell signaling	9644S	1:1000
Acetyl-CoA Carboxylase antibody	Cell signaling	3662S	1:1000
Anti-mouse IgG, HRP-linked Antibody	Cell signaling	7076S	1:10000
Anti-rabbit IgG, HRP-linked antibody	Cell signaling	7074S	1:10000
BNIP3 Antibody	Cell signaling	3769S	1:1000
Cyclophilin B (D1V5J) Rabbit monoclonal antibody	Cell signaling	43603S	1:1000
LC3A/B (D3U4C) antibody	Cell signaling	12741S	1:1000
p70 S6 Kinase antibody	Cell signaling	9202S	1:1000
Phospho-4EBP1 (Thr37/46) rabbit monoclonal antibody	Cell signaling	2855S	1:750
Phospho-p70 S6 Kinase (Thr389) antibody	Cell signaling	9205S	1:750
β-Actin (8H10D10) Mouse monoclonal antibody	Cell signaling	3700S	1:1000
β-Tubulin (9F3) Rabbit monoclonal antibody	Cell signaling	2128S	1:1000
NT5E/CD73 (D7F9A) Rabbit mAb	Cell signaling	13160S	1:1000

E-Cadherin (24E10) Rabbit mAb	Cell signaling	3195S	1:1000
Goat anti-Rabbit IgG (H+L) Cross-Adsorbed Secondary Antibody, Alexa Fluor 568	Invitrogen™	A11011	1:400
Alexa Fluor™ 568 Phalloidin	Invitrogen™	A12380	1:100
Alexa Fluor™ 647 Phalloidin	Invitrogen™	A22287	1:100
Anti-monomeric Keima-Red mAb (Monoclonal Antibody)	MBL	M126-3M	1:1000
OAT Polyclonal antibody	Proteintech	17089-1-AP	1:200
ALDH1B1 Polyclonal antibody	Proteintech	15560-1-AP	1:200

**Dyes**		
Alexa Fluor™ 568 Phalloidin	Thermo Fisher	A12380
HCS LipidTOX™ Deep Red Neutral Lipid Stain, for cellular imaging	Thermo Fisher	H34477
JC-1 Dye (Mitochondrial Membrane Potential Probe)	Thermo Fisher	T3168
Tetramethylrhodamine, Ethyl Ester, Perchlorate (TMRE)	Thermo Fisher	T669
MitoTracker™ Green FM	Thermo Fisher	M7514
BODIPY™ 493/503	Thermo Fisher	D3922
**Drugs**		
MHY1485	Sigma Aldrich	A611700
Torin I	Sigma Aldrich	475991
Dormsomorphin (Compund C)	Sigma Aldrich	P5499
AICAR	Toronto Research Chemicas	5005540001
Leupeptin	Sigma Aldrich	L2884
**Buffers**		
RIPA lysis buffer	Thermo Fisher	J62524-AE
Thermo Scientific™ Halt™ Protease and Phosphatase Inhibitor Cocktail (100X)	Thermo Fisher	78442
Prec Plus Protein Dual Color Standards	Bio-Rad	1610374
10x Tris/Glycine/SDS	Bio-Rad	1610732
2x Laemmli Sample Buffer	Bio-Rad	1610737

Clarity Western ECL Substrate	Bio-Rad	1705061
Seahorse XF base medium, without phenol red, 500 mL.	Aglient	103335-100
Seahorse XF 1.0 M glucose solution, 50 mL	Aglient	103577-100
Seahorse XF 100 mM pyruvate solution, 50 mL	Aglient	103578-100
Seahorse XF 200 mM glutamine solution, 50 mL	Aglient	103579-100
Seahorse XF Calibrant Solution 500 mL	Aglient	100840-000
Seahorse XF Cell Mito Stress Test Kit	Aglient	103010-100
Seahorse XF Mito Fuel Flex Test Kit	Aglient	103260-100
Collagen I, Rat Tail, 100 mg	Corning	354236
Phosphate Buffered Saline pH 7.2, 1X	Quality Bological	114-056-101
Hanks' Balanced Salt Solution 1X	Thermo Fisher	14175-095
Acetic acid	Sigma Aldrich	A6283
Collagenase from Clostridium histolyticum	Sigma Aldrich	C5138
Krebs-Henseleit Buffer Modified	Sigma Aldrich	K3753
Calcium chloride solution	Sigma Aldrich	21115
Sodium bicarbonate	Sigma Aldrich	S6014
Ethylenediaminetetraacetic acid disodium salt solution	Sigma Aldrich	03690
HEPES solution	Sigma Aldrich	H0887
Fetal Bovine Serum	Thermo Fisher	26140079
TMTpro™ 16plex Label Reagent Set	Thermo Fisher	A44520
HiSelect Fe-NTA phosphopeptide enrichment kit	Thermo Fisher	A32992
High Select™ Phosphopeptide Enrichment Kit	Thermo Fisher	A32993
Trypsin Platinum	Promega	VA9000
Urea	Sigma Aldrich	51457
Methanol	Sigma Aldrich	34860
Trifluoroacetic acid	Sigma Aldrich	302031
Acetonitrile solution	Sigma Aldrich	900686
**qPCR Taqman assays**		
Ppia	Thermo Fisher	Mm02342430_g1

Acaca	Thermo Fisher	Mm01304289_m1
Acly	Thermo Fisher	Mm01302282
Fasn	Thermo Fisher	Mm00662319_m1
Scd1	Thermo Fisher	Mm00772290_m1
ND1	Thermo Fisher	Mm04225274_s1
Rnr2	Thermo Fisher	Mm04260181_s1
HK2	Thermo Fisher	Mm00193901_cn
Taqman Fast Advanced Master Mix	Thermo Fisher	4444963
High-Capacity RNA to cDNA kit	Thermo Fisher	4388950
1x Universal Master Mix	Thermo Fisher	4304437
DNeasy Blood and Tissue kit	Qiagen	69504
RNeasy mini kit	Qiagen	74104
**Assay**		
Pierce™ BCA Protein Assay Kit	Thermo Fisher	23225
ATP Determination Kit, 200-1,000 assays	Invitrogen	A22066
Triglyceride (TG) Colorimetric Assay Kit	Elabscience	E-BC-K261-M
Citrate Synthase Assay Kit	Abcam	ab239712
**Other**		
Pierce™ Peptide Desalting Spin Columns	Thermo Fisher	89851
Seahorse XF96 V3 PS Culture Microplates	Aglient	101085-004

Seahorse XFe96 FluxPak	Aglient	102416-100
Mini-PROTEAN® TGX™ Precast Gels (Bio-Rad)	Bio-Rad	4568084
Trans-Blot Turbo Mini NC Transfer Packs	Bio-Rad	1704158

Supplementary References

1 Jang, S. *et al.* Activity and structure of human acetyl-CoA carboxylase targeted by a specific inhibitor. *FEBS Lett* **592**, 2048-2058 (2018). https://doi.org:10.1002/1873-3468.13097

2 Hoffman, N. J. *et al.* Global Phosphoproteomic Analysis of Human Skeletal Muscle Reveals a Network of Exercise-Regulated Kinases and AMPK Substrates. *Cell Metab* **22**, 922-935 (2015). https://doi.org:10.1016/j.cmet.2015.09.001

3 Cho, Y. K. *et al.* STK3/STK4 signalling in adipocytes regulates mitophagy and energy expenditure. *Nat Metab* **3**, 428-441 (2021). https://doi.org:10.1038/s42255-021-00362-2

4 He, Y. L. *et al.* BNIP3 phosphorylation by JNK1/2 promotes mitophagy via enhancing its stability under hypoxia. *Cell Death Dis* **13**, 966 (2022). https://doi.org:10.1038/s41419-022-05418-z

5 Tilokani, L. *et al.* AMPK-dependent phosphorylation of MTFR1L regulates mitochondrial morphology. *Sci Adv* **8**, eabo7956 (2022). https://doi.org:10.1126/sciadv.abo7956

6 Cheng, X., Ma, Y., Moore, M., Hemmings, B. A. & Taylor, S. S. Phosphorylation and activation of cAMP-dependent protein kinase by phosphoinositide-dependent protein kinase. *Proc Natl Acad Sci U S A* **95**, 9849-9854 (1998). https://doi.org:10.1073/pnas.95.17.9849

7 Dietel, E., Brobeil, A., Gattenlohner, S. & Wimmer, M. The Importance of the Right Framework: Mitogen-Activated Protein Kinase Pathway and the Scaffolding Protein PTPIP51. *Int J Mol Sci* **19** (2018). https://doi.org:10.3390/ijms19103282

8 Latorre-Muro, P. *et al.* A cold-stress-inducible PERK/OGT axis controls TOM70-assisted mitochondrial protein import and cristae formation. *Cell Metab* **33**, 598-614 e597 (2021). https://doi.org:10.1016/j.cmet.2021.01.013

9 Zhai, M. *et al.* APN-mediated phosphorylation of BCKDK promotes hepatocellular carcinoma metastasis and proliferation via the ERK signaling pathway. *Cell Death Dis* **11**, 396 (2020). https://doi.org:10.1038/s41419-020-2610-1

10 Park, S. *et al.* Role of the Pyruvate Dehydrogenase Complex in Metabolic Remodeling: Differential Pyruvate Dehydrogenase Complex Functions in Metabolism. *Diabetes Metab J* **42**, 270-281 (2018). https://doi.org:10.4093/dmj.2018.0101

11 Hornbeck, P. V., Zhang, B., Murray, B., Kornhauser, J. M., Latham, V. & Skrzypek, E. PhosphoSitePlus, 2014: mutations, PTMs and recalibrations. *Nucleic Acids Res* **43**, D512-520 (2015). https://doi.org:10.1093/nar/gku1267
