# Supplementary material for: A spatial map of hepatic mitochondria uncovers functional heterogeneity shaped by nutrient-sensing signaling
Source: Nat Commun. 2024 Feb 28;15:1799. doi: 10.1038/s41467-024-45751-9 (PMC10902380; doi:10.1038/s41467-024-45751-9)
Supplement: Supplementary file 3 — Description of Additional Supplementary Files [file 41467_2024_45751_MOESM3_ESM.docx]

DESCRIPTION OF ADDITIONAL SUPPLEMENTARY FILES DOCUMENT

# Supplementary Movies :

**Supplementary Movie S1.** Confocal z-stack (15 μm) of fixed PP region showing mitochondria (green; mitoDendra2) and the cell’s outline (red; actin). scale bar: 10 μm.

**Supplementary Movie S2.** Confocal z-stack (15 μm) of fixed PC region showing mitochondria (green; mitoDendra2) and the cell’s outline (red; actin). scale bar: 10 μm.

**Supplementary Movie S3.** FIB-SEM and volume rendering of PP mitochondria

**Supplementary Movie S4.** FIB-SEM and volume rendering of PC mitochondria

# Supplementary Data :

**Supplementary Data 1.** Differentially expressed proteins and mitochondrial proteome.

**Supplementary Data 2.** Raw phosphoproteome dataset.

**Supplementary Data 3.** Phosphoproteomics dataset normalized to protein abundance.

**Supplementary Data 4.** Global phosphoproteomics system (GPS) analysis.
